# (2,2′-Bipyridine-κ^2^ *N*,*N*′)bromido(1,4,7-trithia­cyclo­nonane-κ^3^ *S*,*S*′,*S*′′)ruthenium(II) hexa­fluoridophosphate

**DOI:** 10.1107/S1600536811002662

**Published:** 2011-01-26

**Authors:** José A. Fernandes, Filipe A. Almeida Paz, Ana I. Ramos, Teresa M. Santos, Susana S. Braga

**Affiliations:** a Department of Chemistry, University of Aveiro, CICECO, 3810-193 Aveiro, Portugal

## Abstract

The title compound, [RuBr(C_10_H_8_N_2_)(C_6_H_12_S_3_)]PF_6_ or [RuBr(bpy)([9]aneS_3_)]PF_6_ ([9]aneS_3_ is 1,4,7-trithia­cyclo­nonane and bpy is 2,2′-bipyridine), exhibits a very similar octahedral coordination geometry for the Ru^2+^ atom to that of its [RuCl(bpy)([9]aneS_3_)]^+^ analogue, with only the chloride ligand being substituted by a bromide ligand. The presence of a PF_6_^−^ anion (alongside with the coordinated bromide ligand) promotes the existence of an extensive network of weak C—H⋯*X* (*X* = F, Br) inter­actions.

## Related literature

For general background to the cytotoxic activity of compounds with the {Ru[9]aneS_3_} moiety, see: Bratsos *et al.* (2008 ▶); Serli *et al.* (2005 ▶). For isotypic compounds based on the [RuCl(bpy)([9]aneS_3_)]^+^ cation, see: Serli *et al.* (2005 ▶); Goodfellow *et al.* (1997 ▶); Fernandes *et al.* (2010 ▶). For previous work from our research group on the use of related compounds, see: Marques, Braga *et al.* (2009 ▶); Marques, Santos *et al.* (2009 ▶).
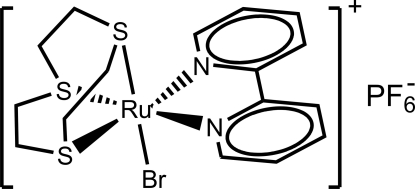


## Experimental

### 

#### Crystal data

[RuBr(C_10_H_8_N_2_)(C_6_H_12_S_3_)]PF_6_
*M* *_r_* = 662.47
Monoclinic, 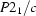

*a* = 12.0660 (7) Å
*b* = 13.4377 (8) Å
*c* = 13.3359 (8) Å
β = 98.446 (3)°
*V* = 2138.8 (2) Å^3^
*Z* = 4
Mo *K*α radiation
μ = 3.03 mm^−1^
*T* = 150 K
0.16 × 0.12 × 0.10 mm

#### Data collection

Bruker APEXII X8 KappaCCD diffractometer
Absorption correction: multi-scan (*SADABS*; Sheldrick, 1998 ▶) *T*_min_ = 0.643, *T*_max_ = 0.752
38626 measured reflections
5736 independent reflections
4989 reflections with *I* > 2σ(*I*)
*R*_int_ = 0.035

#### Refinement

*R*[*F* ^2^ > 2σ(*F* ^2^)] = 0.022
*wR*(*F* ^2^) = 0.046
*S* = 1.06
5736 reflections
271 parameters
H-atom parameters constrained
Δρ_max_ = 0.52 e Å^−3^
Δρ_min_ = −0.63 e Å^−3^

### 

Data collection: *APEX2* (Bruker, 2006 ▶); cell refinement: *SAINT-Plus* (Bruker, 2005 ▶); data reduction: *SAINT-Plus*; program(s) used to solve structure: *SHELXTL* (Sheldrick, 2008 ▶); program(s) used to refine structure: *SHELXTL*; molecular graphics: *DIAMOND* (Brandenburg, 2009 ▶); software used to prepare material for publication: *SHELXTL*.

## Supplementary Material

Crystal structure: contains datablocks global, I. DOI: 10.1107/S1600536811002662/cv5041sup1.cif

Structure factors: contains datablocks I. DOI: 10.1107/S1600536811002662/cv5041Isup2.hkl

Additional supplementary materials:  crystallographic information; 3D view; checkCIF report

## Figures and Tables

**Table 1:** Hydrogen-bond geometry (Å, °)

*D*—H⋯*A*	*D*—H	H⋯*A*	*D*⋯*A*	*D*—H⋯*A*
C1—H1⋯Br1^i^	0.95	2.90	3.6274 (19)	135
C8—H8⋯F1^ii^	0.95	2.42	3.039 (2)	122
C11—H11*A*⋯F1^iii^	0.99	2.41	3.292 (2)	149
C15—H15*A*⋯Br1^iv^	0.99	2.84	3.7627 (18)	156
C16—H16*A*⋯F6^v^	0.99	2.41	3.170 (2)	133

Symmetry codes: (i) 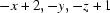; (ii) 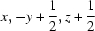; (iii) 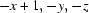; (iv) 
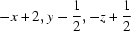
; (v) 
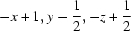
.

**Fig. 1.**
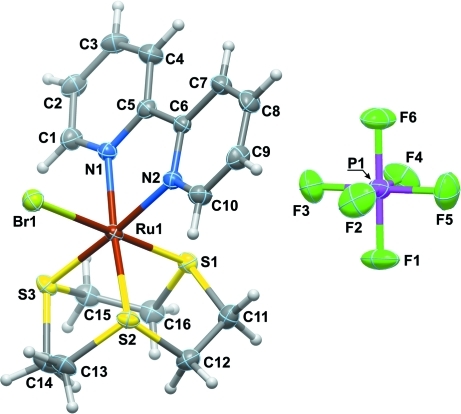
Asymmetric unit of the title compound, with non-hydrogen atoms represented as displacement ellipsoids drawn at the 70% probability level and hydrogen atoms as small spheres with arbitrary radii. The labelling scheme is provided for all non-hydrogen atoms.

**Fig. 2.**
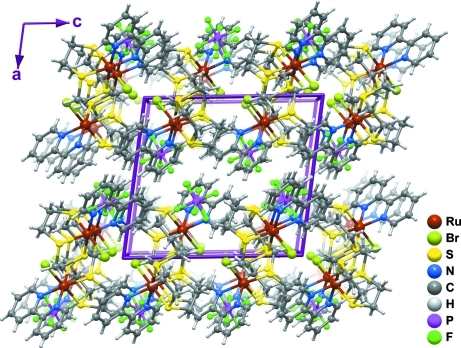
Crystal packing of the title compound viewed in perspective along the b axis.

## supplementary crystallographic information

### Comment

The cytotoxic potential of ruthenium coordination complexes containing 1,4,7-trithiacyclononane ([9]aneS_3_) is under study since 2005 (Bratsos *et al.*, 2008; Serli *et al.*, 2005). We have investigated the cytotoxicity of the [RuCl(glycinate)([9]aneS_3_)] complex on human osteosarcoma and breast cancer cells (Marques, Santos *et al.*, 2009), and the antimicrobial properties of the [RuCl(1,10-phenanthroline)([9]aneS_3_)]Cl complex and its β- and permethylated β-cyclodextrin inclusion compounds (Marques, Braga *et al.*, 2009). We are currently focused on complexes with ([9]aneS_3_)Ru while bearing *N*,*N*-chelated 2,2'-bipyridine (bpy). Recently, we have successfully isolated the monohydrate form of the [RuCl(bpy)([9]aneS_3_)]NO_3_ compound (Fernandes *et al.*, 2010). We wish to report here the structure of [RuBr(bpy)([9]aneS_3_)]PF_6_, isolated as a secondary product from a crystallization batch containing (among other entities) KBr.

The asymmetric unit of the title compound is composed of a whole [RuBr(bpy)([9]aneS_3_)]^+^ cation and a charge-balancing PF_6_^-^ anion. The cation of the title compound shares striking similarities with its [RuCl(bpy)([9]aneS_3_)]^+^ analogue (Serli *et al.*, 2005; Goodfellow *et al.*, 1997; Fernandes *et al.*, 2010), with comparable bond lengths and angles of the coordination environments of Ru^2+^. The difference resides in the substitution of the chlorido ligand by a bromido one, with the Ru1—Br1 distance being enlongated to 2.5720 (2) Å.

The crystal packing is governed by the need to fill the available space and an overall minimization of the interionic distances (Figure 2). Nevertheless, a handful of weak hydrogen bonding interactions is present, namely C—H groups (both aromatic and methylene) interacting  with Br and F of neighbouring ions (not shown; see Table 1 for details). Indeed, the existence of several C—H···F interactions seems to be the structural reason for the absence of the disorder typically associated with the PF_6_^-^ anion.

### Experimental

Chemicals were purchased from commercial sources and were used as received without purification.

Solid KBr (0.0945 g, 79.4 µmol, Sigma-Aldrich) was added to an aqueous solution (2 ml) of γ-cyclodextrin (0.130 g, 101 µmol, Wacker). The resulting solution was poured into a sample holder containing [RuCl([9]aneS_3_)bpy]PF_6_ (0.0248 g, 40.1 µmol). The mixture was magnetically stirred for 1 h at 60 °C. The total volume was then increased by adding 2 ml of distilled water and the temperature was also raised to 70 °C. The mixture was allowed to stir for another hour, after which it was syringe-filtered (nylon, 0.2 µm). The clear solution was allowed to crystallize by slow cooling to ambient temperature inside a sealed container. Orange crystals of the title compound were formed and isolated after two days.

### Refinement

Hydrogen atoms bound to carbon were placed at their idealized positions and were included in the final structural model in riding-motion approximation with C—H = 0.95 Å (aromatic C—H) and 0.99 Å (—CH_2_—). The isotropic thermal displacement parameters for these atoms were fixed at 1.2×*U*_eq_ of the respective parent carbon atom.

### Crystal data

**Table:**

[RuBr(C_10_H_8_N_2_)(C_6_H_12_S_3_)]PF_6_	*F*(000) = 1304
*M**_r_* = 662.47	*D*_x_ = 2.057 Mg m^−^^3^
Monoclinic, *P*2_1_/*c*	Mo *K*α radiation, λ = 0.71073 Å
Hall symbol: -P 2ybc	Cell parameters from 9897 reflections
*a* = 12.0660 (7) Å	θ = 2.6–30.4°
*b* = 13.4377 (8) Å	µ = 3.03 mm^−^^1^
*c* = 13.3359 (8) Å	*T* = 150 K
β = 98.446 (3)°	Block, orange
*V* = 2138.8 (2) Å^3^	0.16 × 0.12 × 0.10 mm
*Z* = 4	

### Data collection

**Table:**

Bruker APEXII X8 KappaCCD diffractometer	5736 independent reflections
Radiation source: fine-focus sealed tube	4989 reflections with *I* > 2σ(*I*)
graphite	*R*_int_ = 0.035
ω and φ scans	θ_max_ = 29.1°, θ_min_ = 3.6°
Absorption correction: multi-scan (*SADABS*; Sheldrick, 1998)	*h* = −16→13
*T*_min_ = 0.643, *T*_max_ = 0.752	*k* = −14→18
38626 measured reflections	*l* = −18→15

### Refinement

**Table:**

Refinement on *F*^2^	Primary atom site location: structure-invariant direct methods
Least-squares matrix: full	Secondary atom site location: difference Fourier map
*R*[*F*^2^ > 2σ(*F*^2^)] = 0.022	Hydrogen site location: inferred from neighbouring sites
*wR*(*F*^2^) = 0.046	H-atom parameters constrained
*S* = 1.06	*w* = 1/[σ^2^(*F*_o_^2^) + (0.0166*P*)^2^ + 1.1306*P*] where *P* = (*F*_o_^2^ + 2*F*_c_^2^)/3
5736 reflections	(Δ/σ)_max_ = 0.002
271 parameters	Δρ_max_ = 0.52 e Å^−^^3^
0 restraints	Δρ_min_ = −0.63 e Å^−^^3^

### Special details

**Table:**

Geometry. All e.s.d.'s (except the e.s.d. in the dihedral angle between two l.s. planes) are estimated using the full covariance matrix. The cell e.s.d.'s are taken into account individually in the estimation of e.s.d.'s in distances, angles and torsion angles; correlations between e.s.d.'s in cell parameters are only used when they are defined by crystal symmetry. An approximate (isotropic) treatment of cell e.s.d.'s is used for estimating e.s.d.'s involving l.s. planes.
Refinement. Refinement of *F*^2^ against ALL reflections. The weighted *R*-factor *wR* and goodness of fit *S* are based on *F*^2^, conventional *R*-factors *R* are based on *F*, with *F* set to zero for negative *F*^2^. The threshold expression of *F*^2^ > σ(*F*^2^) is used only for calculating *R*-factors(gt) *etc*. and is not relevant to the choice of reflections for refinement. *R*-factors based on *F*^2^ are statistically about twice as large as those based on *F*, and *R*- factors based on ALL data will be even larger.

### Fractional atomic coordinates and isotropic or equivalent isotropic displacement parameters (Å^2^)

**Table:**

	*x*	*y*	*z*	*U*_iso_*/*U*_eq_	
Ru1	0.833904 (11)	0.100271 (10)	0.308671 (10)	0.01013 (4)	
Br1	0.971376 (14)	0.213830 (13)	0.421622 (13)	0.01640 (5)	
S1	0.71000 (3)	0.00388 (3)	0.20501 (3)	0.01421 (9)	
S2	0.88870 (4)	0.17069 (3)	0.16651 (3)	0.01378 (9)	
S3	0.96835 (3)	−0.02031 (3)	0.29973 (3)	0.01433 (9)	
N1	0.77196 (11)	0.04462 (11)	0.43571 (10)	0.0125 (3)	
N2	0.71002 (12)	0.20371 (11)	0.32732 (10)	0.0126 (3)	
C1	0.81031 (15)	−0.03531 (14)	0.49059 (13)	0.0173 (4)	
H1	0.8766	−0.0668	0.4760	0.021*	
C2	0.75773 (16)	−0.07386 (15)	0.56726 (14)	0.0197 (4)	
H2	0.7866	−0.1313	0.6035	0.024*	
C3	0.66248 (16)	−0.02759 (14)	0.59036 (14)	0.0195 (4)	
H3	0.6240	−0.0534	0.6419	0.023*	
C4	0.62398 (15)	0.05704 (14)	0.53721 (13)	0.0182 (4)	
H4	0.5599	0.0911	0.5534	0.022*	
C5	0.67964 (14)	0.09190 (13)	0.46003 (13)	0.0135 (3)	
C6	0.64656 (14)	0.18211 (13)	0.40071 (13)	0.0128 (3)	
C7	0.55971 (14)	0.24479 (14)	0.41964 (13)	0.0165 (4)	
H7	0.5145	0.2275	0.4696	0.020*	
C8	0.53990 (15)	0.33168 (14)	0.36549 (14)	0.0191 (4)	
H8	0.4811	0.3750	0.3777	0.023*	
C9	0.60684 (15)	0.35527 (14)	0.29292 (14)	0.0186 (4)	
H9	0.5958	0.4156	0.2556	0.022*	
C10	0.68995 (15)	0.28951 (14)	0.27577 (13)	0.0169 (4)	
H10	0.7350	0.3055	0.2253	0.020*	
C11	0.69306 (15)	0.07354 (14)	0.08615 (13)	0.0177 (4)	
H11A	0.6553	0.0307	0.0311	0.021*	
H11B	0.6441	0.1317	0.0922	0.021*	
C12	0.80353 (15)	0.10941 (14)	0.05783 (13)	0.0176 (4)	
H12A	0.7890	0.1569	0.0006	0.021*	
H12B	0.8454	0.0521	0.0355	0.021*	
C13	1.02665 (15)	0.11727 (14)	0.16039 (15)	0.0185 (4)	
H13A	1.0454	0.1271	0.0913	0.022*	
H13B	1.0831	0.1534	0.2083	0.022*	
C14	1.03368 (15)	0.00714 (14)	0.18570 (14)	0.0188 (4)	
H14A	1.1132	−0.0137	0.1976	0.023*	
H14B	0.9952	−0.0315	0.1275	0.023*	
C15	0.88524 (15)	−0.12899 (13)	0.25441 (14)	0.0168 (4)	
H15A	0.9348	−0.1787	0.2289	0.020*	
H15B	0.8544	−0.1593	0.3120	0.020*	
C16	0.78971 (15)	−0.10518 (13)	0.17114 (14)	0.0180 (4)	
H16A	0.7391	−0.1633	0.1596	0.022*	
H16B	0.8199	−0.0915	0.1074	0.022*	
P1	0.34992 (4)	0.16856 (4)	0.13197 (4)	0.01782 (10)	
F1	0.39585 (10)	0.13350 (10)	0.03098 (9)	0.0359 (3)	
F2	0.41610 (11)	0.27096 (10)	0.12942 (10)	0.0384 (3)	
F3	0.45640 (10)	0.11898 (10)	0.19885 (10)	0.0372 (3)	
F4	0.28321 (10)	0.06540 (9)	0.13318 (10)	0.0327 (3)	
F5	0.24266 (10)	0.21698 (10)	0.06555 (10)	0.0367 (3)	
F6	0.30399 (11)	0.20190 (9)	0.23321 (9)	0.0343 (3)	

### Atomic displacement parameters (Å^2^)

**Table:**

	*U*^11^	*U*^22^	*U*^33^	*U*^12^	*U*^13^	*U*^23^
Ru1	0.01007 (7)	0.00943 (7)	0.01135 (7)	0.00047 (5)	0.00314 (5)	−0.00005 (5)
Br1	0.01564 (9)	0.01541 (10)	0.01780 (9)	−0.00099 (7)	0.00133 (7)	−0.00329 (7)
S1	0.0131 (2)	0.0135 (2)	0.0161 (2)	−0.00181 (17)	0.00253 (16)	0.00033 (17)
S2	0.0153 (2)	0.0123 (2)	0.0147 (2)	−0.00110 (17)	0.00545 (16)	0.00033 (17)
S3	0.0128 (2)	0.0124 (2)	0.0185 (2)	0.00123 (17)	0.00418 (16)	0.00010 (18)
N1	0.0130 (7)	0.0127 (8)	0.0120 (7)	0.0009 (6)	0.0023 (5)	−0.0006 (6)
N2	0.0132 (7)	0.0121 (8)	0.0123 (7)	0.0007 (6)	0.0017 (5)	−0.0012 (6)
C1	0.0190 (9)	0.0166 (10)	0.0163 (9)	0.0032 (8)	0.0022 (7)	0.0006 (7)
C2	0.0264 (10)	0.0165 (10)	0.0160 (9)	0.0005 (8)	0.0027 (7)	0.0036 (8)
C3	0.0252 (10)	0.0192 (10)	0.0155 (9)	−0.0027 (8)	0.0073 (7)	0.0021 (8)
C4	0.0184 (9)	0.0188 (10)	0.0183 (9)	0.0003 (8)	0.0061 (7)	−0.0009 (8)
C5	0.0141 (8)	0.0131 (9)	0.0130 (8)	−0.0011 (7)	0.0013 (6)	−0.0028 (7)
C6	0.0118 (8)	0.0142 (9)	0.0122 (8)	−0.0008 (7)	0.0008 (6)	−0.0019 (7)
C7	0.0138 (8)	0.0198 (10)	0.0167 (9)	0.0007 (7)	0.0047 (7)	−0.0016 (8)
C8	0.0175 (9)	0.0193 (10)	0.0202 (9)	0.0074 (8)	0.0021 (7)	−0.0022 (8)
C9	0.0234 (9)	0.0144 (9)	0.0174 (9)	0.0047 (8)	0.0013 (7)	0.0020 (7)
C10	0.0186 (9)	0.0169 (10)	0.0160 (9)	0.0014 (7)	0.0051 (7)	0.0012 (7)
C11	0.0187 (9)	0.0181 (10)	0.0154 (9)	−0.0013 (8)	−0.0011 (7)	0.0017 (8)
C12	0.0206 (9)	0.0185 (10)	0.0137 (9)	−0.0014 (8)	0.0026 (7)	−0.0004 (7)
C13	0.0152 (8)	0.0183 (10)	0.0239 (10)	−0.0026 (8)	0.0093 (7)	−0.0004 (8)
C14	0.0174 (9)	0.0177 (10)	0.0237 (10)	0.0007 (8)	0.0108 (7)	−0.0011 (8)
C15	0.0207 (9)	0.0094 (9)	0.0214 (9)	0.0014 (7)	0.0067 (7)	0.0001 (7)
C16	0.0217 (9)	0.0110 (9)	0.0215 (10)	−0.0013 (7)	0.0036 (7)	−0.0031 (7)
P1	0.0179 (2)	0.0200 (3)	0.0164 (2)	−0.0014 (2)	0.00541 (18)	0.0004 (2)
F1	0.0393 (7)	0.0464 (8)	0.0265 (7)	−0.0168 (6)	0.0197 (5)	−0.0138 (6)
F2	0.0476 (8)	0.0305 (7)	0.0366 (7)	−0.0212 (6)	0.0052 (6)	−0.0047 (6)
F3	0.0240 (6)	0.0488 (8)	0.0377 (7)	0.0119 (6)	0.0007 (5)	0.0029 (6)
F4	0.0318 (7)	0.0226 (6)	0.0474 (8)	−0.0071 (5)	0.0185 (6)	0.0020 (6)
F5	0.0310 (7)	0.0393 (8)	0.0368 (7)	0.0034 (6)	−0.0051 (6)	0.0124 (6)
F6	0.0441 (7)	0.0375 (8)	0.0246 (6)	0.0123 (6)	0.0157 (6)	−0.0023 (6)

### Geometric parameters (Å, °)

**Table:**

Ru1—N1	2.0881 (14)	C7—H7	0.9500
Ru1—N2	2.0826 (14)	C8—C9	1.385 (3)
Ru1—S1	2.2840 (5)	C8—H8	0.9500
Ru1—S2	2.3011 (4)	C9—C10	1.381 (3)
Ru1—S3	2.3083 (5)	C9—H9	0.9500
Ru1—Br1	2.5720 (2)	C10—H10	0.9500
S1—C11	1.8264 (18)	C11—C12	1.516 (2)
S1—C16	1.8451 (18)	C11—H11A	0.9900
S2—C13	1.8255 (18)	C11—H11B	0.9900
S2—C12	1.8432 (18)	C12—H12A	0.9900
S3—C15	1.8236 (19)	C12—H12B	0.9900
S3—C14	1.8499 (17)	C13—C14	1.517 (3)
N1—C1	1.343 (2)	C13—H13A	0.9900
N1—C5	1.362 (2)	C13—H13B	0.9900
N2—C10	1.346 (2)	C14—H14A	0.9900
N2—C6	1.360 (2)	C14—H14B	0.9900
C1—C2	1.381 (2)	C15—C16	1.513 (3)
C1—H1	0.9500	C15—H15A	0.9900
C2—C3	1.381 (3)	C15—H15B	0.9900
C2—H2	0.9500	C16—H16A	0.9900
C3—C4	1.384 (3)	C16—H16B	0.9900
C3—H3	0.9500	P1—F2	1.5938 (13)
C4—C5	1.390 (2)	P1—F5	1.5956 (13)
C4—H4	0.9500	P1—F6	1.5970 (12)
C5—C6	1.470 (2)	P1—F3	1.5980 (13)
C6—C7	1.396 (2)	P1—F1	1.6007 (12)
C7—C8	1.375 (3)	P1—F4	1.6043 (12)
			
N2—Ru1—N1	78.08 (5)	C8—C9—H9	120.6
N2—Ru1—S1	91.91 (4)	N2—C10—C9	122.97 (16)
N1—Ru1—S1	90.43 (4)	N2—C10—H10	118.5
N2—Ru1—S2	97.01 (4)	C9—C10—H10	118.5
S1—Ru1—S2	88.644 (16)	C12—C11—S1	112.87 (13)
N1—Ru1—S3	97.38 (4)	C12—C11—H11A	109.0
S1—Ru1—S3	88.460 (17)	S1—C11—H11A	109.0
S2—Ru1—S3	87.533 (16)	C12—C11—H11B	109.0
N2—Ru1—Br1	86.93 (4)	S1—C11—H11B	109.0
N1—Ru1—Br1	90.81 (4)	H11A—C11—H11B	107.8
S2—Ru1—Br1	89.993 (13)	C11—C12—S2	110.80 (12)
S3—Ru1—Br1	92.811 (13)	C11—C12—H12A	109.5
N1—Ru1—S2	174.97 (4)	S2—C12—H12A	109.5
N2—Ru1—S3	175.45 (4)	C11—C12—H12B	109.5
S1—Ru1—Br1	178.095 (13)	S2—C12—H12B	109.5
C11—S1—C16	100.98 (9)	H12A—C12—H12B	108.1
C11—S1—Ru1	102.38 (6)	C14—C13—S2	113.27 (12)
C16—S1—Ru1	106.28 (6)	C14—C13—H13A	108.9
C13—S2—C12	101.30 (9)	S2—C13—H13A	108.9
C13—S2—Ru1	104.44 (6)	C14—C13—H13B	108.9
C12—S2—Ru1	105.64 (6)	S2—C13—H13B	108.9
C15—S3—C14	99.63 (8)	H13A—C13—H13B	107.7
C15—S3—Ru1	102.88 (6)	C13—C14—S3	111.14 (12)
C14—S3—Ru1	106.86 (6)	C13—C14—H14A	109.4
C1—N1—C5	118.13 (14)	S3—C14—H14A	109.4
C1—N1—Ru1	126.36 (11)	C13—C14—H14B	109.4
C5—N1—Ru1	115.39 (11)	S3—C14—H14B	109.4
C10—N2—C6	118.19 (15)	H14A—C14—H14B	108.0
C10—N2—Ru1	126.09 (11)	C16—C15—S3	113.37 (13)
C6—N2—Ru1	115.69 (11)	C16—C15—H15A	108.9
N1—C1—C2	122.99 (17)	S3—C15—H15A	108.9
N1—C1—H1	118.5	C16—C15—H15B	108.9
C2—C1—H1	118.5	S3—C15—H15B	108.9
C3—C2—C1	118.96 (18)	H15A—C15—H15B	107.7
C3—C2—H2	120.5	C15—C16—S1	110.89 (12)
C1—C2—H2	120.5	C15—C16—H16A	109.5
C2—C3—C4	118.92 (16)	S1—C16—H16A	109.5
C2—C3—H3	120.5	C15—C16—H16B	109.5
C4—C3—H3	120.5	S1—C16—H16B	109.5
C3—C4—C5	119.58 (17)	H16A—C16—H16B	108.0
C3—C4—H4	120.2	F2—P1—F5	90.24 (7)
C5—C4—H4	120.2	F2—P1—F6	90.75 (7)
N1—C5—C4	121.33 (16)	F5—P1—F6	90.06 (7)
N1—C5—C6	115.04 (14)	F2—P1—F3	90.43 (8)
C4—C5—C6	123.61 (16)	F5—P1—F3	179.30 (8)
N2—C6—C7	121.23 (16)	F6—P1—F3	89.74 (7)
N2—C6—C5	115.22 (14)	F2—P1—F1	89.98 (7)
C7—C6—C5	123.49 (15)	F5—P1—F1	90.33 (7)
C8—C7—C6	119.64 (16)	F6—P1—F1	179.17 (8)
C8—C7—H7	120.2	F3—P1—F1	89.86 (7)
C6—C7—H7	120.2	F2—P1—F4	179.36 (7)
C7—C8—C9	119.13 (17)	F5—P1—F4	89.46 (7)
C7—C8—H8	120.4	F6—P1—F4	89.83 (7)
C9—C8—H8	120.4	F3—P1—F4	89.86 (7)
C10—C9—C8	118.78 (17)	F1—P1—F4	89.45 (7)
C10—C9—H9	120.6		
			
N2—Ru1—S1—C11	75.99 (7)	Ru1—N1—C1—C2	−173.00 (14)
N1—Ru1—S1—C11	154.07 (7)	N1—C1—C2—C3	−1.2 (3)
S2—Ru1—S1—C11	−20.99 (6)	C1—C2—C3—C4	−1.2 (3)
S3—Ru1—S1—C11	−108.55 (6)	C2—C3—C4—C5	1.9 (3)
N2—Ru1—S1—C16	−178.51 (7)	C1—N1—C5—C4	−2.1 (2)
N1—Ru1—S1—C16	−100.42 (7)	Ru1—N1—C5—C4	174.23 (13)
S2—Ru1—S1—C16	84.52 (6)	C1—N1—C5—C6	176.53 (15)
S3—Ru1—S1—C16	−3.05 (6)	Ru1—N1—C5—C6	−7.18 (19)
N2—Ru1—S2—C13	161.69 (8)	C3—C4—C5—N1	−0.3 (3)
S1—Ru1—S2—C13	−106.56 (7)	C3—C4—C5—C6	−178.74 (17)
S3—Ru1—S2—C13	−18.04 (7)	C10—N2—C6—C7	2.6 (2)
Br1—Ru1—S2—C13	74.78 (6)	Ru1—N2—C6—C7	−179.04 (13)
N2—Ru1—S2—C12	−91.93 (7)	C10—N2—C6—C5	−174.73 (15)
S1—Ru1—S2—C12	−0.17 (6)	Ru1—N2—C6—C5	3.59 (19)
S3—Ru1—S2—C12	88.35 (6)	N1—C5—C6—N2	2.4 (2)
Br1—Ru1—S2—C12	−178.84 (6)	C4—C5—C6—N2	−179.06 (16)
N1—Ru1—S3—C15	72.45 (7)	N1—C5—C6—C7	−174.92 (16)
S1—Ru1—S3—C15	−17.78 (6)	C4—C5—C6—C7	3.6 (3)
S2—Ru1—S3—C15	−106.49 (6)	N2—C6—C7—C8	−2.1 (3)
Br1—Ru1—S3—C15	163.64 (6)	C5—C6—C7—C8	175.04 (17)
N1—Ru1—S3—C14	176.84 (8)	C6—C7—C8—C9	0.0 (3)
S1—Ru1—S3—C14	86.61 (7)	C7—C8—C9—C10	1.4 (3)
S2—Ru1—S3—C14	−2.10 (7)	C6—N2—C10—C9	−1.2 (3)
Br1—Ru1—S3—C14	−91.97 (7)	Ru1—N2—C10—C9	−179.30 (14)
N2—Ru1—N1—C1	−177.12 (15)	C8—C9—C10—N2	−0.8 (3)
S1—Ru1—N1—C1	91.02 (14)	C16—S1—C11—C12	−64.36 (15)
S3—Ru1—N1—C1	2.51 (15)	Ru1—S1—C11—C12	45.21 (14)
Br1—Ru1—N1—C1	−90.43 (14)	S1—C11—C12—S2	−48.58 (17)
N2—Ru1—N1—C5	6.94 (12)	C13—S2—C12—C11	135.80 (13)
S1—Ru1—N1—C5	−84.92 (12)	Ru1—S2—C12—C11	27.14 (14)
S3—Ru1—N1—C5	−173.42 (11)	C12—S2—C13—C14	−68.39 (15)
Br1—Ru1—N1—C5	93.64 (12)	Ru1—S2—C13—C14	41.20 (15)
N1—Ru1—N2—C10	172.54 (15)	S2—C13—C14—S3	−45.34 (17)
S1—Ru1—N2—C10	−97.42 (14)	C15—S3—C14—C13	133.84 (14)
S2—Ru1—N2—C10	−8.56 (15)	Ru1—S3—C14—C13	27.13 (15)
Br1—Ru1—N2—C10	81.06 (14)	C14—S3—C15—C16	−67.85 (14)
N1—Ru1—N2—C6	−5.62 (12)	Ru1—S3—C15—C16	42.06 (13)
S1—Ru1—N2—C6	84.42 (12)	S3—C15—C16—S1	−47.66 (15)
S2—Ru1—N2—C6	173.28 (11)	C11—S1—C16—C15	135.46 (13)
Br1—Ru1—N2—C6	−97.10 (12)	Ru1—S1—C16—C15	28.95 (13)
C5—N1—C1—C2	2.8 (3)		

### Hydrogen-bond geometry (Å, °)

**Table:**

*D*—H···*A*	*D*—H	H···*A*	*D*···*A*	*D*—H···*A*
C1—H1···Br1^i^	0.95	2.90	3.6274 (19)	135
C8—H8···F1^ii^	0.95	2.42	3.039 (2)	122
C11—H11A···F1^iii^	0.99	2.41	3.292 (2)	149
C15—H15A···Br1^iv^	0.99	2.84	3.7627 (18)	156
C16—H16A···F6^v^	0.99	2.41	3.170 (2)	133

Symmetry codes: (i) −*x*+2, −*y*, −*z*+1; (ii) *x*, −*y*+1/2, *z*+1/2; (iii) −*x*+1, −*y*, −*z*; (iv) −*x*+2, *y*−1/2, −*z*+1/2; (v) −*x*+1, *y*−1/2, −*z*+1/2.
